# Endoscopic removal of a large foreign body retained in the duodenum: A case report

**DOI:** 10.1097/MD.0000000000020857

**Published:** 2020-07-02

**Authors:** Ya-nan Guo, Fang Li, Fu Huang, Tao Yu

**Affiliations:** a Shaanxi University of Traditional Chinese Medicine, Xian-yang, Shaanxi; b Department of spleen and stomach, Shaanxi Traditional Chinese Medicine Hospital, Xi-an, China.

**Keywords:** chopsticks, endoscopic removal, foreign body incarceration

## Abstract

**Introduction:**

This study aimed to present the case of a patient in whom a chopstick, which had been in the duodenum for 10 years, was finally removed by endoscopy. This case was reported because of the long-time retention and noninvasive removal by endoscopy without sedation or complication.

**Patient concerns:**

A 30-year-old male patient with intermittent upper abdominal pain.

**Diagnoses:**

During upper-gastrointestinal (GI) endoscopy, a long-strip foreign body (Fb) was seen in the descending part of the duodenum. An upper-GI barium examination was performed, which revealed a linear Fb in the duodenum cavity. The Fb was >10-cm long. Combined with his history (the patient admitted swallowing a chopstick 10 years ago in a bet), the diagnosis of Fb in the duodenum was confirmed.

**Interventions:**

The Fb was removed from the duodenum cavity by upper-GI endoscopy successfully.

**Outcomes:**

The patient was discharged after the removal of the Fb.

**Conclusion:**

Endoscopic removal and nonoperative management might be feasible in carefully selected patients with a long and old Fb in the duodenum without the need for anesthesia or surgery as well as no occurrence of complications and laceration.

## 1 Introduction

Foreign body (Fb) ingestion is a common emergency. Patients who purposely swallow a true object are typically younger, more often male, and commonly associated with psychiatric illness or drug abuse.^[1]^ The diagnosis often can be made based on the patient's history. A careful physical examination should be done to detect signs of perforation, such as subcutaneous emphysema or severe abdominal pain, as well as nausea and vomiting. Computed tomography (CT) scanning is superior to plain radiography and identifies the Fb in 70% to 100% of patients.^[2]^

Fb in the duodenum is more specific than that in other parts of the upper-gastrointestinal (upper-GI) tract. Li et al, in their series of 1088 cases, noted that only 0.04% of ingested Fbs were lodged in the duodenum.^[3]^ To date, only a few cases have been reported on the removal of large Fbs retained in the duodenum using upper-GI endoscopy. A case of long-term retention of a pencil in the upper-GI tract was reported by Lianjun et al.^[4]^ After 5 years of swallowing the pencil, the patient developed abdominal pain, as well as nausea and vomiting, when the body position changed. The diagnosis of perforation by imaging examination was definite, and surgical treatment was the final choice. The present case was reported because of the long-time retention and noninvasive removal by endoscopy without sedation or complication.

In the past, retained Fbs were usually removed by open laparotomy. Actually, the majority (80%–90%) of Fbs pass spontaneously; only 20% of Fb ingestions require endoscopic removal while surgery is necessary for 1% of them.^[2]^ No epidemiological data were available on the retention time of Fbs in the upper-GI tract, but the time of endoscopic intervention was dictated by the perceived risks of aspiration or perforation; those without evidence of high-grade obstruction or acute distress could be handled less urgently.^[1]^

The patient has provided informed consent for the publication of the case.

## 2 Case report

**Figure 1:**
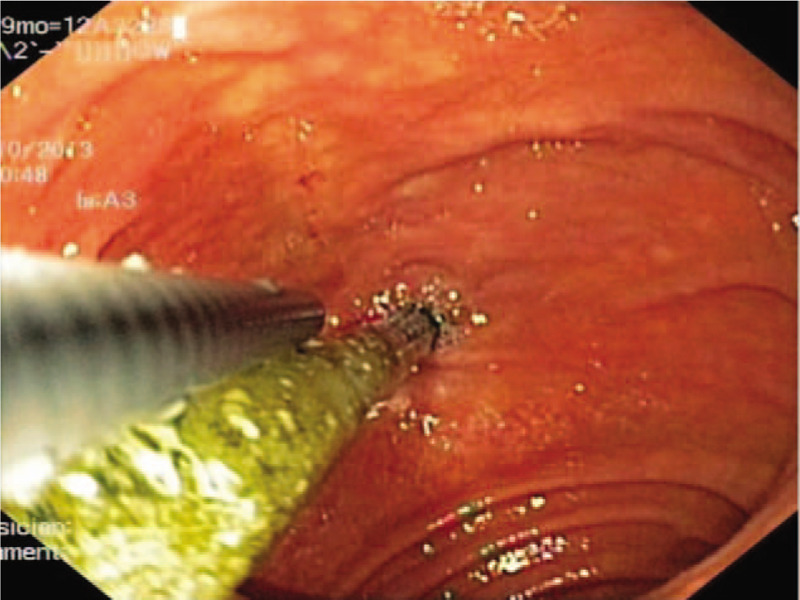
A long strip chopstick was seen in upper gastrontestinal endoscopy at duodenum descending part.

**Figure 2:**
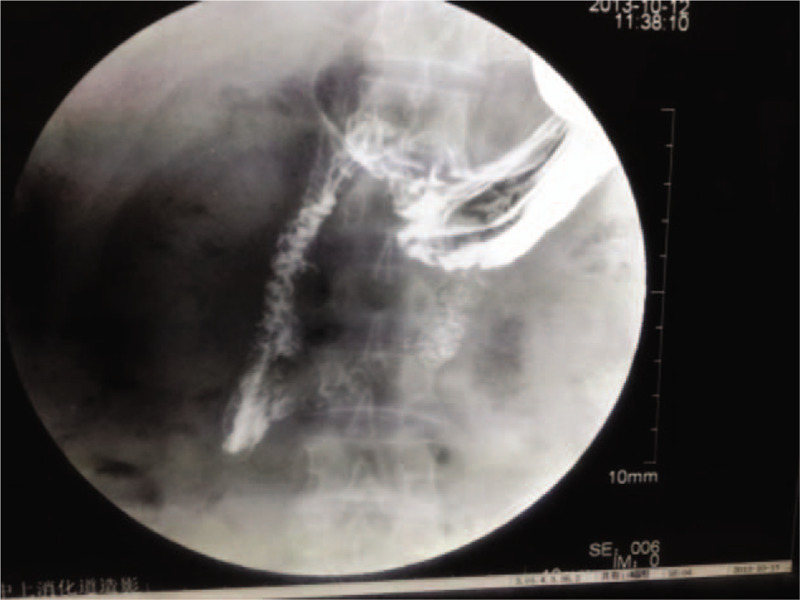
A linear foreign body in the duodenum cavity was seen in an upper-gastrontestinal barium examination.

A 30-year-old male patient with intermittent upper-abdominal pain consulted the department of spleen and stomach in Shaanxi Traditional Chinese Medicine Hospital. During upper-GI endoscopy, a long-strip Fb was observed in the descending part of the duodenum (Fig. 1). No obvious edema or ulceration was found where the Fb was lodged. The length and material of the Fb were difficult to determine. After the surgery, the operator asked for the patient's medical history again in detail. The patient admitted swallowing a chopstick 10 years ago in a bet. The patient's physical examination showed slight tenderness over his epigastrium, and no local abdominal bulge or rebound tenderness. An upper-GI barium examination was performed, which revealed a linear Fb, >10-cm long, in the duodenal cavity (Fig. 2). Combined with the patient's history, the diagnosis of Fb in the duodenum was confirmed.

**Figure 3:**
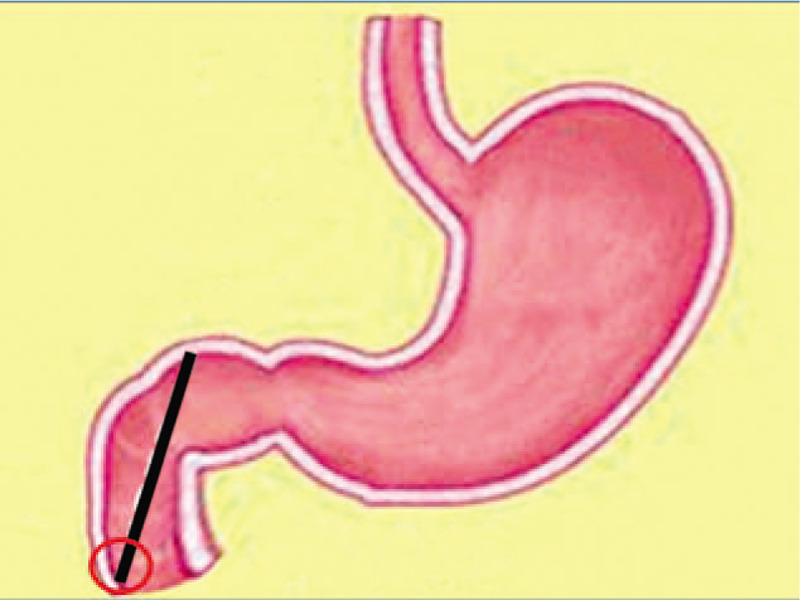
Chopstick stucks vertically, and the procedure was started with the black circle.

**Figure 4:**
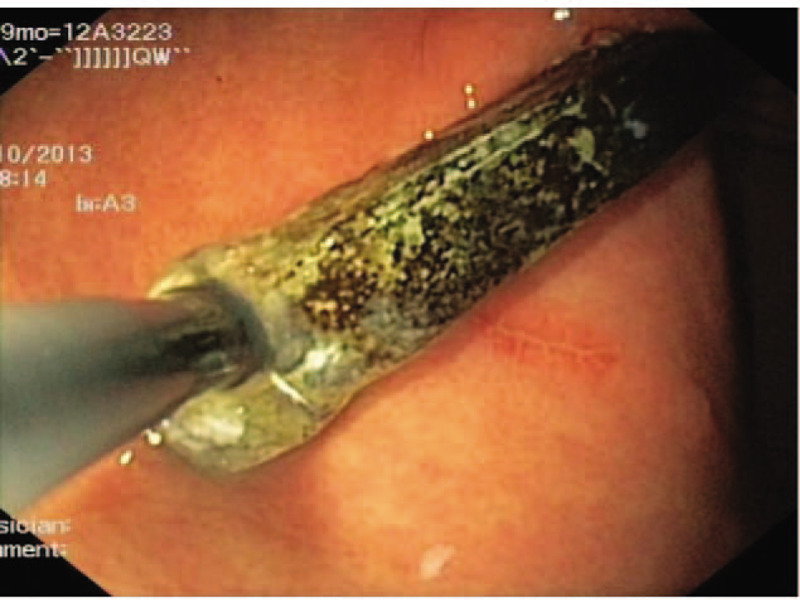
Trying to remove chopstick by endoscopic forceps.

**Figure 5:**
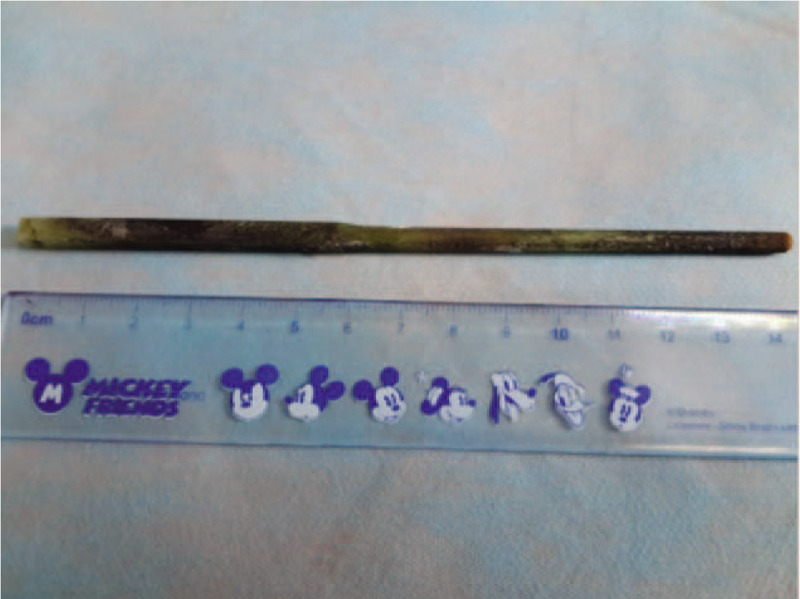
Removal of a 14 cm long chopstick by endoscopic forceps was accomplished successfully.

The final decision was to use a single-channel endoscopy to complete the removal of the Fb. Endoscopic removal of the Fb >10 cm was more likely to fail. Li et al suggested using a double-channel endoscope and a double-snare to remove Fb objects such as chopsticks.^[3]^ Even so, the success rate of removal of Fb >10 cm was still very low at their endoscopy center. The removal of the chopstick was performed in 2 steps in the present case: to avoid sticking, the procedure was started with the black circle (Fig. 3); then, fixing the snare at the distal end of the chopstick, the operator dragged the endoscope to the distal end, whereas the assistant retracted the snare until it became movable. The snare was slid to the near end of the chopstick (Fig. 4). The direction of the chopstick was adjusted according to the field-of-endoscopy vision, maintaining the long axis of the chopstick in line with the esophagus and finally pulling it out (Fig. 5). The patient underwent another endoscopy to ensure no mucosal damage or bleeding and received routine treatment with conventional antacid and stomach-protecting drugs. A physician closely monitored the patient for fever, abdominal pain, melena, and other abdominal signs; repeat CT or endoscopy was performed if necessary. No complications occurred, and the symptoms of epigastric pain disappeared after the patient was treated with conventional antacid and stomach-protecting drugs.

## 3 Discussion

The common complications of Fb in the GI tract, such as mucosal laceration, bleeding, pyrexia, and perforation, were fortunately not present, which was the most important reason for the 10-year retention of the Fb in this case. The duodenum had an oblique diameter of only 7.3 to 10.4 cm.^[5]^ Therefore, the chopstick in this case stuck vertically, and the anatomical characteristics of the duodenum made it difficult for the giant Fb (≥10 cm) to pass through the physiological bending part. The angle between the second and third parts of the duodenum is so quite small and the smooth muscle structure of the duodenum wall results in inferior lateral and longitudinal elasticity, allowing movement only in the form of peristalsis.

Imaging examination played a key role in the choice of Fb treatment. Some scholars suggested that for patients with Fb impaction for >24 hours or with an endoscopic indication for more difficult treatment and higher risk, preoperative CT examination needed to be performed. It could not only clearly identify but also assess the risk. In addition, barium examination is also a good choice. In this case, an Fb >10-cm long was found in the second part of the duodenum; the corresponding duodenum was stiff, with no sign of barium leakage. Moreover, the patient showed no obvious signs of peritonitis or melena. Therefore, a diagnosis of perforation was not considered.

The risk of an Fb in the digestive tract is related to its shape and size, as the mortality and the risk of perforation increase with the size of these objects, leading to peritonitis, abscess formation, inflammatory mass formation, obstruction, fistulae, and hemorrhage.^[3]^ Endoscopic intervention is the criterion standard technique used for the removal of fb from GI tract,^[6]^ and postoperative complications of surgical treatment included wound infection, pneumonia, and formation of biliary fistula,^[7]^ but in some cases, fb ingestion is followed by life-threatening complications, for which surgical intervention is essential.

## 4 Conclusions

To sum up, Fb ingestion should be kept in mind in a patient admitted with abdominal pain. Clinical suspicion and patient history may help to make the diagnosis clear or avoid unnecessary surgery. Moreover, a flexible endoscope is the diagnostic and therapeutic method of choice for fb in the duodenum with strong operability, less trauma, and fewer complications.

## Author contributions

**Conceptualization:** Tao Yu, Ya-nan Guo.

**Data curation:** Fang Li, Fu Huang.

**Formal analysis:** Tao Yu.

**Investigation:** Fang Li, Fu Huang.

**Resources:** Tao Yu.

**Software:** Ya-nan Guo.

**Validation:** Tao Yu.

**Visualization:** Tao Yu.

**Writing – original draft:** Ya-nan Guo,Fu Huang, Fang Li, Tao Yu.

**Writing – review & editing:** Ya-nan Guo, Fang Li, Tao Yu.
